# Application of radiofrequency ablation in duodenal mucosal reconstruction

**DOI:** 10.1055/a-2134-7209

**Published:** 2023-08-21

**Authors:** Zhengqi Li, Biao Zhou, Nianrong Zhang, Siqi Wang, Hua Meng

**Affiliations:** Department of General Surgery and Obesity and Metabolic Disease Center, China-Japan Friendship Hospital, Beijing, China

A 63-year-old woman was diagnosed with type 2 diabetes 8 months ago and was scheduled for duodenal mucosal reconstruction. The patient took metformin orally to control blood sugar. Before the operation, fasting blood glucose was 7.0 mmol/L (15.7 mmol/L 2 hours after a meal), and glycosylated hemoglobin was 7.1 %. A single-channel flexible endoscope (EVIS GIF-N170; Olympus, Tokyo, Japan) was introduced into the horizontal part of the duodenum, and the Endoscopic Catheter (Barrx Channel; Medtronic, Minneapolis, USA) was inserted into the biopsy channel of the endoscope ( Fig. 1 ). Radiofrequency ablation of the duodenal mucosa was performed, starting from the horizontal part of the duodenum, while the endoscope was gradually withdrawn ( Fig. 2 ). By rotating the endoscope and the catheter, all four quadrants of the duodenal mucosa were ablated ( Fig. 3 ). Overall, a 13-cm length of duodenal mucosa was ablated without bleeding or perforation ( Fig. 4 , Video 1 ).

**Fig. 1:**
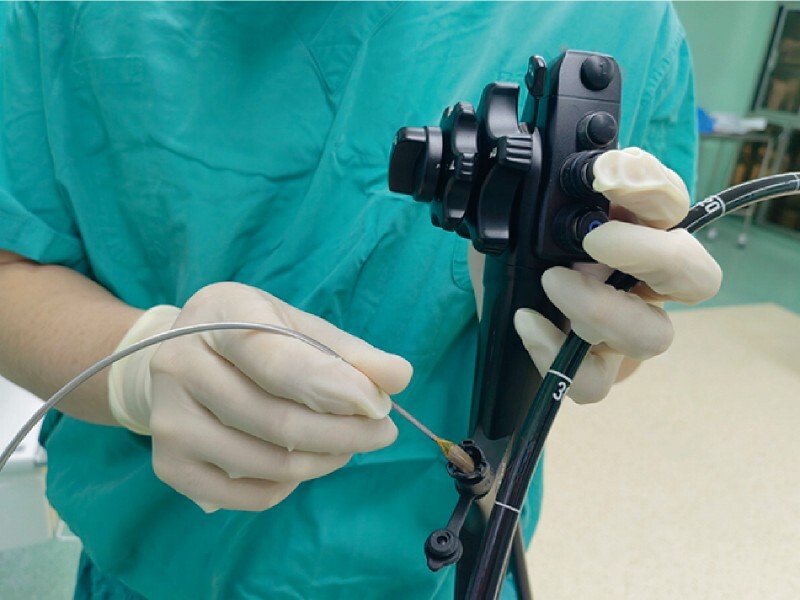
The endoscopic catheter was inserted into the biopsy channel of the endoscope.

**Fig. 2:**
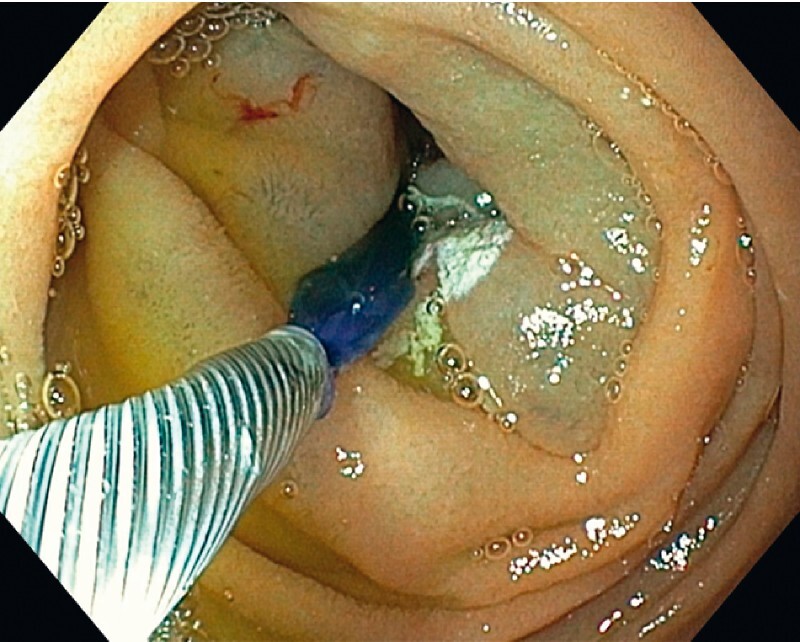
Radiofrequency ablation of the duodenal mucosa was performed from the horizontal part of the duodenum.

**Fig. 3:**
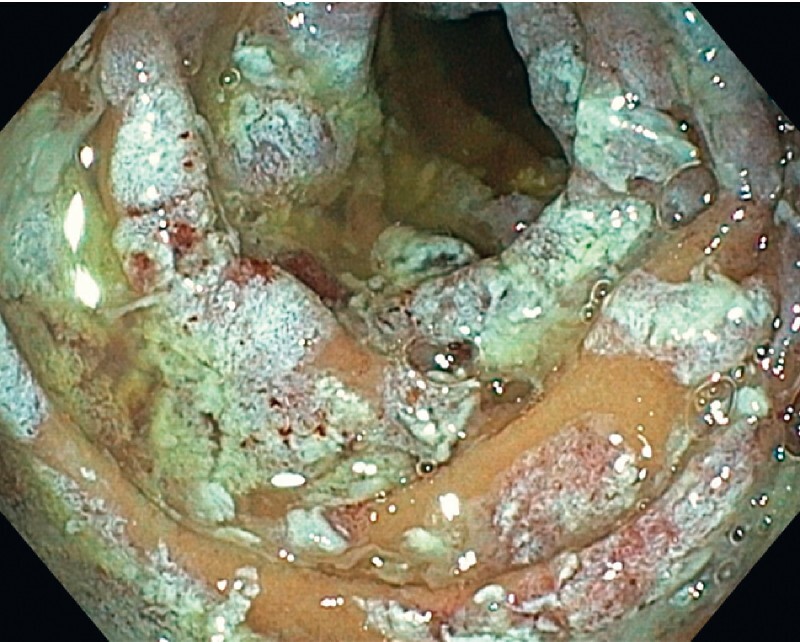
By rotating the endoscope and the catheter, all four quadrants of the duodenal mucosa were ablated.

**Fig. 4:**
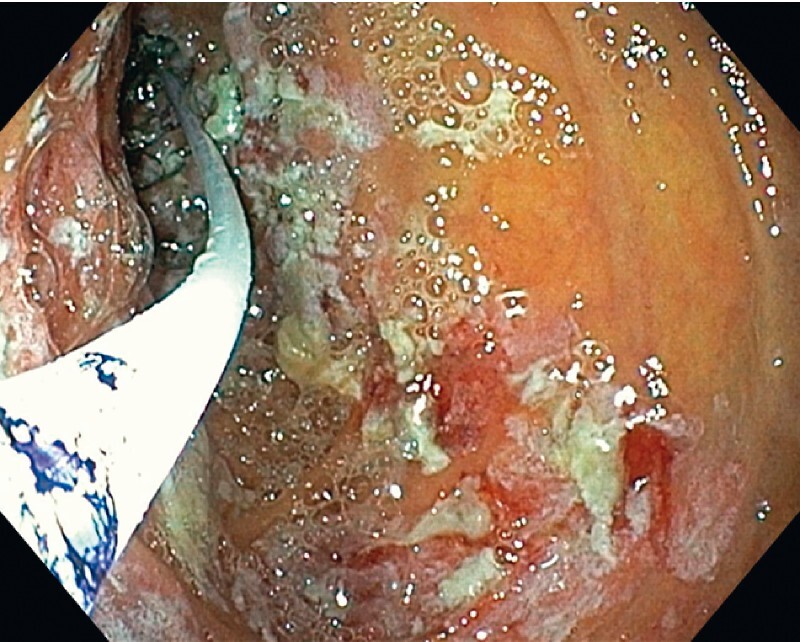
A 13-cm length of duodenal mucosa was ablated without bleeding or perforation.

**Video 1** Application of radiofrequency ablation in duodenal mucosa reconstruction.


The key points of this operation were: 1) the planned radiofrequency ablation sessions targeted the descending and horizontal parts of the duodenum; 2) the power used was 12 J/cm ^2^ , 48 W; 3) possible adverse events were hemorrhage and perforation; 4) the sedation was general anesthesia (tracheal intubation); 5) the duration of the procedure was 92 minutes.

During the follow-up period of 1 month, the patient stopped taking hypoglycemic drugs, fasting blood glucose decreased to 6.0 mmol/L (11.8 mmol/L 2 hours after a meal), and glycosylated hemoglobin was 6.2 %. The patient did not experience any discomfort.

Duodenal mucosal reconstruction is a catheter-based endoscopic procedure designed to lower blood sugar by altering the surface of the duodenal mucosa 1 2 . The characteristics of the radiofrequency ablation system are: 1) precise ablation control, which effectively reduces the risk of complications; 2) prediction of treatment effect, which limits damage to normal tissues 3 4 . We therefore applied this system to the duodenum to achieve a surgical effect similar to that of duodenal mucosal reconstruction.

Endoscopy_UCTN_Code_TTT_1AO_2AN

## Notes

Endoscopy E-Videos

https://eref.thieme.de/e-videos
*E-Videos* is an open access online section of the journal *Endoscopy* , reporting on interesting cases and new techniques in gastroenterological endoscopy. All papers include a high-quality video and are published with a Creative Commons CC-BY license. Endoscopy E-Videos qualify for HINARI discounts and waivers and eligibility is automatically checked during the submission process. We grant 100% waivers to articles whose corresponding authors are based in Group A countries and 50% waivers to those who are based in Group B countries as classified by Research4Life (see: https://www.research4life.org/access/eligibility/ ). This section has its own submission website at https://mc.manuscriptcentral.com/e-videos
